# Supplementary material for: Acute febrile illness surveillance using TaqMan Array Cards in two urban health facilities, Monrovia, Liberia, December 2018–March 2020
Source: PLoS Negl Trop Dis. 2026 Feb 9;20(2):e0013961. doi: 10.1371/journal.pntd.0013961 (PMC12885267; doi:10.1371/journal.pntd.0013961)
Supplement: Attachment — Submitted filename: Response to reviewers.docx [file pntd.0013961.s001.docx]

Dear reviewers and PLOS NTD editors,

Thank you very much for the feedback and comments on our manuscript (PNTD-D-24-01227), we feel that they strengthen our manuscript considerably. In our revision and below, we attempted to address and respond to the review and provide more detail where necessary.

In summary, the following the changes were also made:

- We included table 3 that provide more detail on patient characteristics for select TAC +ve detections (namely *Plasmodium* spp., dengue, *Rickettsia* spp., and *S. pneumoniae*).
- Tables 4 and 5 (formerly Tables 3 and 4) now present stratified analyses by site and age groups (2-14 versus 15+ years) with row percentages.
- The target and sequences of the assays included on our custom TAC have been removed from the appendix as these have been published previously along with assay performance characteristics (<https://journals.asm.org/doi/full/10.1128/jcm.02257-15>).
- An author summary has been added as a section between the abstract and background.
- Throughout the revision, we clarify our objectives, describe the target population in more detail, assess imported infections, provide more detail on and expand on undifferentiated fever in regards to the enrollment criteria and related analyses.
- Affiliations include cities.
- “Formal” added to the informed consent process under ethical statement.

Our specific responses to reviewer comments:

1. Table 1. Please indicate the numbers of inpatients and outpatients

- A row for in-patients was added.

2. The analyses presented in Tables 3 and 4 are pooled data from Redemption Hospital and Star of the Sea Health Centre. However, the target patients and disease severity between the hospital and health center, and likely the risk factors are different (as shown in Table 1). A stratified analysis by health facilities should be better performed.

- Tables for a stratified analysis by health facility and age groups were added as tables 4 and 5.

3. Tables 3 & 4. Please present the row percentages (TAC positive as the main outcome)

- Percentages were changed to row, thank you for pointing out this oversight.

4. Tables 3 & 4. Please explain why some estimates (e.g. animal exposure, travel history) were not presented.

- Per the methods (line 205), only variables with p<0.1 in bivariate analysis were included in the final multivariate model in addition to age and gender.

5. Figure 2, the colours for Plasmodium spp and Lassa fever were too similar.

- Noted, *Plasmodium spp.* was changed to purple.

**Methods**

Reviewer #1:

You conducted a multivariate model to assess risk factors for any Tac +ve result. There wasn't a study aim that aligned with this analysis, and I think it would be helpful to describe the rationale for this analysis - presumably given the positive results were overwhelmingly malaria, essentially you are presenting risk factors for a positive falciparum PCR assay.

- Thank you for this comment. We consider this project and analysis to be exploratory as it provides an overview and insight into febrile-causing pathogens circulating at two sentinel sites in urban Monrovia. Certainly, one of the objectives is also to characterize patients with TAC +ve results and describe our implementation of AFI surveillance, the multivariate models contribute to the characterization as well as provide insights for AFI implementation at these sites. To make this more explicit, we added to the objectives in the background (lines 125-128) and discussion (line 463-466).
- Yes, we fully agree, the majority of TAC +ve detections were from malaria and more so for Redemption Hospital than Star of the Sea. There were insufficient data unfortunately to more fully explore non-malaria TAC detections in stratified multivariate analysis and note this in the discussion and limitations (lines 448-451). We do think this is noteworthy data to present given the differences by site, age groups, and presenting symptoms and certainly deserving of additional exploration.

2. I recommend explicitly stating the pathogens tested for using the Tac card - I don't think the assay names in the appendix (eg "PhHV" or "Sudan" or "MS") are really sufficient.

- Noted, we reworked Table 1 to be more specific including in a footnote and referenced a manuscript detailing the TAC development of nearly identical design and assays in the methods that also provides detail.

Reviewer #2: Lines 107 – 108:

The population of the study is insufficiently described. Patients attending the two health care facilities were seen from a “variety” of demographic and geographic regions from Liberia is too vague. And why highlighting this variety whereas the aim of the study was to explore causes of fever and circulating pathogens in urban Monrovia?

- Thank you for this comment, we attempted to reword and clarify to avoid vagueness and agree ‘“variety” of demographic and geographic regions’ is inadequate. Patients could be residents (part time or full time) in urban Monrovia and still travel outside of Montserrado County. Given the proximity of both health facilities to major transit points, they have high patient volumes and may receive patients who reside outside of Monrovia but may be visiting (and could have been infected while in Monrovia or introduce infectious pathogens). We reworded this sentence in attempt to better reflect this.

This sentence suggests also that these two health care facilities have a spatial representativeness of the population in Liberia. Geographical provenance of patients should be described more precisely for the two health care facilities, in particular their attractivity area.

- Certainly the sentence was not meant to imply a spatial representativeness of the two health care facilities for all of Monrovia; we consider these sites as sentinel sites for AFI surveillance and hopefully the revision throughout more accurately reflects this. Approximate patient population served by the two health facilities (lines 145, 151) are noted but more detailed catchment data was unavailable and beyond the scope for this analysis (although noting that only 3% of AFI patients reported any travel history beyond Montserrado County).

Lines 114 - 115: “… a projected 2014 population of nearly 35000 in an area of just over 0.5 km²” gives 70000 inhabitants/km² that seems very high. Please check both population and area.

- We agree, this is a very high density. We rechecked both data, the population cited is consistent with the 2022 census (<https://lisgis.gov.lr/document/LiberiaCensus2022Report.pdf>). The acreage is frequently cited (or its metric conversions) and appears to be based on a Liberian government report as well (<https://openjicareport.jica.go.jp/pdf/11965852_01.pdf>). This calculated density is also consistent with what has been reported (<https://www.iut.nu/wp-content/uploads/2017/03/Liberian-Housing-Profile.pdf>). As West Point is adjacent to the sea and is prone to flooding, the area is subject to change and acreage may be based on a “habitable area” estimate.

Line 117: At patient enrollment, was a self-questionnaire used or was it self-administered? Please, clarify this point.

- AFI project staff screened patients for eligibility using a standardized form (line 164) and additional detail to the enrollment screening process has been added to the methods to clarify.

Line 120: Please, could you recall symptoms of Ebola and what happened when a patient had these. Was it an exclusion criterion? However, this pathogen is searched with TAC. I don’t understand.

- Thank you for this comment, additional details were added in the enrollment sub-section for more clarity (lines 158-162). Ebola screening was a blanket routine process at these health facilities (a positive Ebola screen would trigger heightened facility precautions but not necessarily an exclusion criterion for the AFI project), and AFI project staff tailed on this facility screening process to identify potential febrile patients to enroll.

Lines 123 – 127: During the pseudo-random sampling some days of the week were chosen and other were not, why?

- The days of the week were chosen for logistical reasons. Health facility staff noted Mondays to be the busiest given patients could not be seen over the weekend. Wednesday was chosen to allow spacing for laboratory processing of the specimens. We added additional detail to clarify (lines 143-144).

Line 135: What is exactly a self-report of fever? Feeling hot? Chills reported within the previous 7 days.

- Yes, that is correct. A self-report of fever is based on patient (or parent/guardian) recall in the past 7 days of feeling hot (“skin hot” per the Liberian context) or chills (line 185).

Line 135: There were no spatial criteria of exclusion. Did you mean that patients coming from outside Liberia were also included?

- Yes, that is correct, there was no spatial criteria of exclusion. Potentially, full-time or part-time residents of Monrovia could have traveled from outside Montserrado County or Liberia. In addition, we did not want to exclude visitors residing outside Monrovia who may have become infected while in Monrovia or may be importing infectious illnesses to Monrovia. We attempted to clarify in the methods (line 195).

Line 138: “… a documented measured axillary, oral, or rectal temperature ≥ 37.5°C…” is one of the inclusion criteria. It is admitted that an axillary measure underestimate temperature of 0.5°C versus rectal measure. Please mention this bias of measurement.

Lines 137 – 139: In my opinion, the case definition is very sensitive and/or with some level of uncertainty as fever could be declared. I think that TAC negative tests could be also done wrongly for patients with no fever. The case definition itself could explain also the high percentage of negative TAC tests discussed lines 320 - 323, in particular at the Star of the Sea health care centre.

- Thank you for this comment, yes, the case definition is indeed sensitive. As an initial exploratory analysis, we wanted the project to be as sensitive as possible to capture a wide range of patients who possibly had a febrile-causing pathogen. For future AFI screenings in Monrovia or Liberia, revision of the enrollment criteria would most certainly be revisited. The large numbers of negative TAC tests are also consistent with other AFI surveillance projects (line 418) and highlights the difficult in identifying etiologic causes of undifferentiated febrile illnesses.
- We are in agreement that axillary measurements may underestimate temperatures and added to the limitations (line 399), but 83% of AFI patients still had a measured fever at enrollment. These were also sick patients seeking healthcare for an illness and anti-pyretic usage (overall a third of AFI patients reportedly took medication prior to AFI enrollment) potentially lowering their temperatures could also not be ruled out. We also note that measured fever was not a significant predictor for a TAC +ve detection for all but 1 of the site and age group strata. Some other alternative explanations for the high percentage of negative TAC tests could also be other febrile-causing pathogens not on TAC, medication usage from self-treatment or serial/concurrent healthcare seeking, and/or time from symptom onset to specimen collection being beyond the ability of TAC to detect as noted in the discussion and limitations.

Line 141: “Exclusion criteria included…” is a little bit confusing.

- Thank you, the sentence was revised.

Line 145: “… with a clear source of infection”. I don’t understand well what does it mean? Patients with no clinical cause of fever identified despite a diagnostic approach based on clinical signs and/or paraclinical tests could be an additional criterion of the case definition? At this point, the reader may wonder about the objective of the study which was initially to explore the causes of fever and the pathogens circulating in the urban area of Monrovia. It is necessary to clarify the objective of the study and the causes of fever that were explored.

- Thank you for this feedback. The intention of this criterion during the screening process was to exclude possible non-infectious causes of fever or etiologic causes that TAC could not test for. We revised the enrollment sub-section (line 192) to note the specific questions administered during the screening process. Patients with no clinical cause of fever identified could certainly be eligible to be enrolled. The need to clarify our objective(s) is well noted here and in other reviewer comments, and we revised in our manuscript for clarity accordingly.

**Results**

Reviewer #1: 1. Do you think the +ve falciparum PCR assays in the Tac card represent the cause of illness? Presumably with such a high proportion positive, many will have asymptomatic infection. What was the correlation with more conventional malaria tests?

- This is certainly an important point, we can not definitively conclude that +ve TAC detections are the cause of fever, especially for malaria given its high number of detections and high prevalence. We added this to the limitations as well (line 445). There is an additional analysis forthcoming that compares the TAC malaria results with the facility malaria results that demonstrate a high correlation.

2. Given the high proportion with negative results from the Tac card, were there any other conventional diagnostic tests performed (eg blood or urine culture), and are these results available?

- Unfortunately no, the only corresponding test results available are from the facility malaria tests which a forthcoming analysis will detail a comparison. We did note in the results and table 1 the facility malaria test results and general alignment with TAC malaria detections. Overall, there was a high level of agreement between the facility malaria rapid diagnostic tests and TAC results with differences due to the lengthier detection windows from malaria RDTs.

Reviewer #2: Line 217: Please, inform the percentage of patients > 15 years.

- Thank you for this, Table 1 has been revised for this oversight to include age groups as well as in the results (line 280).

Lines 221 – 222: What were the causes of admissions at the Redemption hospital?

- Unfortunately, it was beyond the scope of this project to examine the causes of admissions among enrolled patients at Redemption Hospital. We presumed that hospitalization meant a more serious illness and possibly a greater likelihood of TAC detection, but this does not appear to be evident in bivariate or multivariate analysis (Tables 4a, 5a).

Lines 240 – 244: For the other pathogens detected than Plasmodium, were there imported cases?

- For both the AFI patients who reported any travel history within the past 30 days and for the 6 AFI patients residing outside of Montserrado County all TAC +ve detections were malaria. We added additional sentences in the results of this detail (lines 305-308) as well as in table 3.

Line 258: In table 3, I am not sure for the OR of sex as the sum of TAC negative patients according to gender is more than 439. Please check for this.

- Thank you for this observation, tables 4 and 5 have been substantially revised for a stratified analysis and this may not be applicable.

Tables and figures

Figure 1
Figure 1 seems to be a screen capture. Some pathogens have abbreviations, MS2, 18S, CCHF, PhHV, 16S, and should be informed. Please, could you improve this figure and render it clearer.
Some pathogens seem not detected by TAC as antimicrobial resistance, tuberculosis, Borrelia, mycobacteria, influenza, Q fever, VRS, adenovirus. The epidemiological relevance of these last in the AFI surveillance should be discussed.

- Thank you for this comment. Figure 1 has been edited for clarity and more detail added in the footnote. It is beyond our scope to be able to discuss the epidemiological relevance of other febrile illnesses beyond those tested by our TAC, but we do note that our findings are only based on pathogens selected for inclusion on TAC (line 469), a limitation. We also note that TAC can be specifically configured for diarrheal and ILI causing pathogens (line 98). *Coxiella burnetti* (Q fever) is also included as a detectable pathogen on our TAC (Figure 1).

It would be interesting in knowing the distribution of main pathogens (Plasmodium, Salmonella, Streptococcus…) among age groups (< and ≥ 15 years).

- We certainly agree that further characterization of the TAC +ve detections would be interesting and have debated what data to prioritize given the multipathogen aspect using TAC for AFI surveillance. Given an issue with the small numbers and the remote possibility of identifying patients, we included a table for *Plasmodium* spp., dengue, Rickettsia, and *S. pneumoniae* detections by some characteristics as these each at least 10 detections (Table 3).

As pregnant exhibit 45 and 4.4% of female according to health care facilities, why not include this variable in the univariate analysis.

- Thank you for noting this oversight, pregnant status should have been included in bivariate (and for a multivariate model of Redemption Hospital AFI patients 15+ years) analyses. We have made this revision in table 4.

For missing and unknown answers, how did they analyse?

- For enrolled AFI patients, demographic data (i.e., gender, age, education) did not have missing or unknown answers except when noted in Table 1 for pregnancy status and occupation. Calculated percentages include the missing. Other variables collected such as animal exposure, travel history, and symptoms were based on yes/no boxes or check boxes. We note, however, all but 24 patients reported another symptom besides fever (line 289) and assume that AFI project staff administered questions to patients for all the symptoms as instructed and received responses. Our modest numbers during AFI enrollment days allowed AFI staff sufficient time to complete thorough questionnaires as well.

Table 4
Abdominal pain is significant for positive TAC tests whereas cough is significant for negative one. Does it mean that TAC could potentially detect more pathogens with a digestive tropism than pathogens with a respiratory one?

- Thank you for noting this. With the reworked stratification of tables 4 and 5, this situation seems to apply for table 5a (2-14 years olds at Redemption Hospital) but with cough not being significant in multivariate analysis. We would be very hesitant to make definitive conclusions but do think it’s an observation deserving of future exploration particularly among young children. We also note that TAC focusing on respiratory pathogens have been developed and utilized previously but beyond our scope and resources.

**Conclusions**

Reviewer #1: 1. Among limitations, it is also worth noting that PCR assays are not the most sensitive test for many pathogens. For example, blood culture using automated systems is the reference test for bacteremia (eg typhoid) and diseases such as leptospirosis are complicated with different sample types (eg urine) or assays (eg MAT serology) preferred later in the illness.

- Thank you for this comment, we agree that PCR assays such as TAC may be limited in their ability to detect some of the pathogens. We also included this in the discussion and limitations (lines 452).

2. There are major differences in the proportion with a TAC detection between sites - could you discuss this in more detail? Might it relate to severity of illness and/or prevalence of respiratory viral infection?

- Thank you for this, yes, we agree that there is a difference in TAC detections between the sites and attempted to expand in the results and table 3. We also stratified our analyses in tables 4 and 5 by age group (<15 and 15+ years) and health facility which provide more detail. The difference may be due to the underlying patient demographics or self selection bias for health seeking at the two facilities. It is beyond the scope of this analysis to explore the prevalence of respiratory viral infections but do note that in Table 1 the difference in presenting symptoms. Furthermore in multivariate analysis hospitalization (an indicator of illness severity) at Redemption Hospital was not significant for those ages 15+ years and not significant in bivariate analysis for those ages 2-14 years.

Reviewer #2: Line 282: If appropriate, dengue cases should be discussed according to imported cases Cf. my comments for lines 135 and 240 – 244.

- Thank you for noting this, all +ve detections for patients with any travel history or residence outside of Montserrado County were for malaria. We added this finding to the results (lines 305-308) and explicitly in the context for dengue and Rickettsia in the discussion (lines 362-365).

Lines 295 – 296: Efficiency of TAC could be discussed with that of some RDT (malaria, meningitis…). Also, what is the diagnostic value of TAC (sensitivity, specificity…) according to the main pathogens encountered in Liberia? Is fever the best sign to prescribe a TAC test versus other symptoms or combination of symptoms like influenza-like illness in example.

- Thank you for this comment. It was beyond our resources and scope to be able to evaluate our TACs sensitivity and specificity. We do note a previous publication on a nearly identically designed TAC’s diagnostic characteristics <https://journals.asm.org/doi/full/10.1128/jcm.02257-15> (line 230). As TAC is fully customizable, the performance of TAC depends on the underlying pathogens and assays selected to include as well as their targets. The pathogens we selected are known etiologic cause of undifferentiated fever; patients with a measured or recent fever were appropriate to test for our version of TAC. We also can not rule out possible co-infection with other pathogens not tested by TAC. We also note that symptoms consistent with ILI (i.e., cough) still resulted in +ve TAC detections.

Lines 313 - 316: The distinction between pathogen detection by site should be discussed also regarding a selection bias as the recruitment of an hospital is different of that of a health care centre.

- We certainly agree that patient characteristics may be different between a referral hospital and primary care health centre, and our data supports this. We attempted to describe these differences throughout the results and in the discussion. However, our AFI project is an initial exploratory analysis at two sentinel health care sites in urban Monrovia; because of this, we would not necessarily characterize the differences as a selection bias but rather as differences noteworthy to describe as it provides context for consideration of the interpretation of the results as well as a consideration for other surveillance efforts and future exploration. We acknowledge that differences in detected pathogens may be due to differences in underlying patient characteristics, health seeking behaviors, or the surrounding communities (lines 383-397).

Line 318: Animal exposure is searched in the previous 30 days and is significant for positive TAC. Which pathogens were expected to be detected? How this variable was measured? The (self-?) administered questionnaire would be interesting in supplementary material to see how questions were formulated. I think a memory bias should be also discussed for this variable.

- With the stratified analysis by age and health facility (tables 4 and 5), any animal exposure in multivariate analyses was only significant for <15 years among AFI patients at Redemption Hospital. Any animal exposure was based on questions on type of animal and type of contact (i.e., touch, slaughter, delivery, shear, or other) including for bushmeat. Additional detail was added to the methods (line 256). The rationale for probing on animal contact was not so much to explore a direct relationship with a specific TAC detectable pathogen but more as a proxy for exposure to rural environments/outdoors (and likely higher risk for tick-borne, mosquito-borne illnesses or zoonoses) and SES.

Line 321: Please, see my comments for lines 137 – 139 and line 145.

- Yes, thank you for the feedback. We have noted this and made revisions that hopefully further clarifies.

Lines 346 – 348: You can add that another cause of non-representativity of the overall population is the pseudo-random sampling as pointed in lines 123 – 127. As the aim of the study was to explore causes of fever and circulating pathogens in urban Monrovia, it is more a spatial representativity which should be reached and discussed here.

- Yes, thank you for this comment here and elsewhere. We do note this in the limitations (line 62) and our revisions attempt to clarify throughout the manuscript that we consider the health facilities as sentinel sites, and this is an initial exploratory project.

**Editorial and Data Presentation Modifications?**

**Summary and General Comments**

Reviewer #1: nil additional

Reviewer #2: The study population should be better described as it concerned inhabitants in Monrovia. The case definition seems very sensitive and need additional explanations and discussion.

- Thank you for this feedback. We attempted to clarify the populations at the two sites throughout and emphasize that we consider the two sites as sentinel locations (rather than representative of all of Monrovia). As noted previously, we consider this AFI project as exploratory and wanted to utilize a sensitive case definition. This has been added to the methods and discussion (lines 352-360).

Introduction
Lines 71 – 73: I don’t agree with the fact that complex and expensive laboratory diagnostics are not widely available as there are a large offer of rapid diagnostic tests even if their efficiency need more study, cf. Reference Bouzid D et al. Rapid diagnostic tests for infectious diseases in the emergency department. Clin Microbiol Infect. 2021 Feb;27(2):182-191. doi: 10.1016/j.cmi.2020.02.024. Please could you discuss the role of TDRs in the epidemiological surveillance and link their use to that of TAC?

- Thank you for this comment. Our intention was to note the testing limitations at public healthcare facilities for the general patient population in Liberia. We recognize that it may be inaccurate to generalize to all low-income settings and certainly “complex and expensive” is very context dependent. We attempted to refine in our revision (lines 94-96). As for the role of RDTs in epidemiological surveillance, it is beyond the scope of this manuscript. We do, however, support the use of surveillance data such as from this AFI project, in identifying and prioritizing potential diagnostics (including RDTs) to implement in healthcare facilities (line 368). A future analysis comparing facility malaria RDT versus TAC malaria detections is in process.

Lines 82 – 84: Could you quantify the impact of febrile illnesses on health consultations and hospital admissions at Monrovia/Liberia instead of “substantial burden” that remains vague.

- This is well noted. The previous lines note that 42% of patient consultations in Liberia were for malaria (2013, the latest year of data) with an estimated 1.6 million patients being suspected of malaria (i.e., febrile) (2021). We also added that more than 77,000 severe malaria patients were hospitalized. We believe these data collectively do support an argument that malaria is a substantial burden on Liberia’s healthcare system.

Line 99: “… implementation of AFI sentinel surveillance using TAC at two health”, the sentence is not finished.

- Thank you, this incomplete sentence has been addressed.

We hope our responses and revisions have fully addressed reviewer concerns. If additional clarifications are needed, please do not hesitate to reach out. Again, we appreciate the consideration and effort by reviewers, we feel the analysis and manuscript have been strengthened. We hope our manuscript is accepted for publication.

Thank you,

Dr. Terrence Lo and co-authors
