# Supplementary material for: Acute febrile illness surveillance using TaqMan Array Cards in two urban health facilities, Monrovia, Liberia, December 2018–March 2020
Source: PLoS Negl Trop Dis. 2026 Feb 9;20(2):e0013961. doi: 10.1371/journal.pntd.0013961 (PMC12885267; doi:10.1371/journal.pntd.0013961)
Supplement: Attachment — Submitted filename: Response to reviewers 11.25 (B).docx [file pntd.0013961.s002.docx]

7 November 2025

Dear reviewers and PLOS NTD editors,

Thank you very much for the feedback and comments on our manuscript (PNTD-D-24-01227), we have attempted to respond and clarify in our manuscript where possible to the feedback below (in blue). In addition, we:

- Embedded the tables and figure titles within the body of the manuscript
- Uploaded figures as separate graphic files, including figure 1 which was erroneously not included in the previous submission
- Removed the highlighting from some of the citations
- Identified a point of contact for data access that was not a co-author

Thank you, and please do let us know if any additional clarifications are needed.

The Authors have improved the manuscript and addressed many of the comments. However, the Authors are expected to address all the remaining criticisms by the two Reviewers. In particular, please clarify the study limitations and conclusions (Reviewers #1 and #2). In additional to these comments, please address:

1. Abstract, the results for the multivariable analysis were reported for Redemption Hospital only. While it is reasonable to emphasize more on Redemption Hospital which has a larger sample size, it may be more complete to add a brief overall description for Star of the Sea Health Centre, or highlight key similarities or differences (e.g. without presenting the specific estimates).

Thank you for the comment. We included the TAC detection by facility (lines 44-45), added the facility for detection for *N. meningitidis* and Lassa virus (the detected pathogens with epidemic potential) (lines 49-50), and already had included the non-malaria TAC detection by facility (line 51). We hope these sufficiently highlight the key differences between the two facilities in the abstract.

**Journal Requirements:**

1) Please provide an Author Summary. This should appear in your manuscript between the Abstract (if applicable) and the Introduction, and should be 150-200 words long. The aim should be to make your findings accessible to a wide audience that includes both scientists and non-scientists. Sample summaries can be found on our website under Submission Guidelines:
https://journals.plos.org/plosntds/s/submission-guidelines#loc-parts-of-a-submission

An author summary was included on the “clean” version of the manuscript (but not on the “tracked changes” version) on the previous submission, our apologies for the oversight. It is included on lines 68-82.

2) Please upload a copy of Figure Figure 1 which you refer to in your text on page 11.. Or, if the figure is no longer to be included as part of the submission please remove all reference to it within the text.

Figure 1 has been uploaded to the submission repository along with figures 2 and 3. This was also a previous oversight, thank you.

3) Tables should not be uploaded as individual files. Please remove these files and include the Tables in your manuscript file as editable, cell-based objects. For more information about how to format tables, see our guidelines:
https://journals.plos.org/plosntds/s/tables

This is well noted and our apologies for thid oversight as well. All tables and figure titles have been inserted in the main body of the manuscript. Thank you.

**Reviewers' comments:**

Reviewer's Responses to Questions

**Key Review Criteria Required for Acceptance?**

As you describe the new analyses required for acceptance, please consider the following:

**Methods:**

-Are the objectives of the study clearly articulated with a clear testable hypothesis stated?
-Is the study design appropriate to address the stated objectives?
-Is the population clearly described and appropriate for the hypothesis being tested?
-Is the sample size sufficient to ensure adequate power to address the hypothesis being tested?
-Were correct statistical analysis used to support conclusions?
-Are there concerns about ethical or regulatory requirements being met?

Reviewer #1: Nil - previous suggestions have been addressed.

Reviewer #2: It is a descriptive study based on a sentinel surveillance with two sites sufficiently depicted. This design allows to answer to the main aim of the study, to explore causes of undifferentiated fever and their causal infectious agents. Populations attending the two sentinel sites are sufficiently described.
For reason of limited resources, the authors used a pseudo-random sample from the two sentinel sites. As they performed a multivariate logistic regression after stratification on age and sentinel site, they have to discuss the probably insufficient statistical power of the study in limitations, in discussion.

Thank you for the feedback and comments, they are appreciated. We fully acknowledge that our sampling framework is not representative of neither the febrile patient population seeking care at these two facilities or the urban Monrovia population overall. We also agree that the lack of representativeness limits the interpretation of our data including the descriptive demographics and multivariate models. We added some additional text to the limitation to make this point more explicit along with the already existing acknowledgement that interpretation of our data should be limited only to those who were enrolled and tested (lines 512-515).

To be more precise, did in-patient already hospitalized at their inclusion or were they admitted after retaking temperature upon entrance into health facilities?

Only Redemption Hospital had in-patients (line 146), and patients may have potentially been enrolled while they were hospitalized since they could have been admitted when AFI project staff were not working (i.e., Sunday night, Tuesday night). The fever inclusion criteria still would have been met for enrollment, however (i.e., measured or report of fever within the past 7 days, lines 176-178) including a potential re-measurement of temperature. We hope this is clear and answers the question?

The pragraph about ethical considerations is clear.

**Results:**

-Does the analysis presented match the analysis plan?
-Are the results clearly and completely presented?
-Are the figures (Tables, Images) of sufficient quality for clarity?

Reviewer #1: nil - previously addressed.

Reviewer #2: The analysis match the analysis plan and the results are complete.

Thank you.

**Conclusions:**

-Are the conclusions supported by the data presented?
-Are the limitations of analysis clearly described?
-Do the authors discuss how these data can be helpful to advance our understanding of the topic under study?
-Is public health relevance addressed?

Reviewer #1: In the limitations section:
Line 398-399: "Our data also suggests that Star of the Sea functioned sentinel surveillance site for non-malaria detections." I think this sentence needs revision for clarity. Perhaps '...functioned as a sentinel..."

We changed the text as suggested, thank you for noting this necessary edit.

Line 438-439 "Utilizing whole blood also may not be an ideal specimen type to detect some pathogens (e.g. blood culture for Typhoid fever)" Are you suggesting bone marrow aspirate is the preferred sample type, or that PCR is less sensitive than culture?

Thank you for this comment. Our intention in the limitations is to note that while TAC can be utilized multi-pathogen detections, whole blood may not be the ideal specimen type or the gold standard for some. We hope that this is clearer with the addition of “gold standard” (line 494). It is beyond our analysis to compare specimen types but we do note that TAC testing performance on a nearly identical layout have been summarized elsewhere (line 223).

In your conclusions:
I appreciate that you identify that you have insufficient non-malarial TAC positive participants, but it would be useful to flag that an analysis to identify those at high risk of testing positive for non-malarial pathogens would be helpful. Given the high cost of TAC-array cards, it would be ideal to know which group of patients to deploy them in.

This is well noted, we completely agree that further exploration of non-malaria causes of fever should be undertaken and added a sentence in the discussion (lines 459-460) and conclusion (lines 520-521) to make it more explicit with the existing text (“need to identify other circulation pathogens beyond malaria” (lines 396), “findings suggest adolescents, commonly reported non-febrile symptoms, animal exposures may be relevant variables to increase detection yields” (lines 456-458). Given that there were only 29 non-malaria detections, more detailed analyses was not possible. We do note, however, that non-malaria detections were significantly higher at Star of the Sea, a smaller health centre (lines 312-313) and table 3 presents patient demographics, hospitalization status, symptoms, and other variables for dengue, rickettsia, and *S. pneumoniae,* the most common non-malaria detections.

Reviewer #2: In the conclusion, this seems too far going writing performing TAC can serve as an outbreak indentification tool in the article whereas it seems more a warning tool. Indeed, a pseudo-random sampling is not sufficiently exhaustive to make currently outbreak detection with sentinel site surveillance. A complete surveillance system must have a clear and acurate case definition for each infectious disease to reach this goal and the alone criteria based on fever is too restrictive. I leave the authors explaining the positioning of TAC.

Thank you for this comment. We fully agree and acknowledge that the timeframe of this analysis is too short to establish baseline trends (assuming a broad definition of an outbreak being above baseline) and the sampling framework was not representative (and was not intended to be as our objective was pathogen discovery, lines 123-125, 519).

We counter that for many of the AFI TAC pathogens, especially the high consequence ones, only 1 positive detection was necessary to meet the threshold for an “outbreak” (e.g., EVD, MVD, Lassa fever, Yellow fever, dengue, etc.) per Liberia’s IDSR guidelines and others as well. AFI TAC positive detections of Lassa virus, *N. meningitidis*, and dengue virus (and arguably Liberia’s first human detection of rickettsia) do constitute outbreaks of these diseases. We added some additional text in the discussion (lines 412-414) and conclusion (lines 522-523) to clarify.

For discussion, i remind to discuss the probably insufficient statistical power of the study in limitations.

Yes, thank you for your comment, and we hope our edit and response above is sufficient.

In limitations, the authors wrote (line 439) "Although we attempted to enroll febrile patients during active infection". As it is depicted in the paragraph Patient enrollment, it seems difficult to catch infection at active phase on the alone fever criteria.

Thank you for this comment. There are several possibilities for AFI TAC not detecting any pathogens: (1) Enrolled patients were outside their active infection period (i.e., incorrectly recalled their onset date of fever, temperature measurement errors) (2) Pathogen causing the fever was not tested for on our AFI TAC (3) Pathogen detection was beyond the detection time for TAC. We did take steps to address (1) such as retaking auxillary temperature measurements (line 161) but without introducing additional testing (2) and (3) could not be evaluated. We do note that our AFI detections are in-line with other AFI surveillance efforts utilizing TAC in other settings (lines 464-465).

Even though it is not part of the objectives, it would be interesting in having some cost data relating to TAC.

We completely agree, the cost of surveillance using TAC would be interesting and is an important consideration in resource constrained settings. The costs could have wide variation depending on the negotiated amounts, institutional agreements, and availability of human resources to support which is unfortunately beyond the scope of our analysis.

The remaining of the conclusion is relevant for public health.

**Editorial and Data Presentation Modifications?**

Use this section for editorial suggestions as well as relatively minor modifications of existing data that would enhance clarity. If the only modifications needed are minor and/or editorial, you may wish to recommend “Minor Revision” or “Accept”.

Reviewer #1: nil

Reviewer #2: Minor modification
Line 289: the majority instead of themajority

Thank you for catching this typo, it has been corrected.

**Summary and General Comments:**

Use this section to provide overall comments, discuss strengths/weaknesses of the study, novelty, significance, general execution and scholarship. You may also include additional comments for the author, including concerns about dual publication, research ethics, or publication ethics. If requesting major revision, please articulate the new experiments that are needed.

Reviewer #1: Thank you for the opportunity to review your revised manuscript. Overall, my comments/queries have been addressed. I have a few remaining/new minor comments (listed above)

Reviewer #2: It is an interesting study about the difficult questioning of the "unobvious" causes of febrile illness. It is based on a sentinel surveillance with two sites that differ in their population coverage. In this way, there is potentially a large variety of causal agents. The study asks the place of tests like TAC in warning, surveillance and their cost-effective yield.
The limited resources led to use a pseudo-random sampling that questioned the representativness and the statistical power of the study whereas a multivariate logistic regression is performed.
The discussion is complete and conclusion is relevant for the challenge in detection of emergent and re-emergent infectious disease.

Thank you very much for both of your considerable insights and thorough reviews, it is very much appreciated!
